# Simultaneous Mass Spectrometric Detection of Proteins of Ten Oilseed Species in Meat Products

**DOI:** 10.3390/foods11142155

**Published:** 2022-07-20

**Authors:** Johannes Spörl, Karl Speer, Wolfgang Jira

**Affiliations:** 1 Department of Safety and Quality of Meat, Max Rubner-Institut (MRI), E.-C.-Baumann-Straße 20, 95326 Kulmbach, Germany; johannes.spoerl@mri.bund.de; 2 Faculty of Chemistry and Food Chemistry, Technical University of Dresden, Helmholtzstraße 10, 01069 Dresden, Germany; karl.speer@chemie.tu-dresden.de

**Keywords:** foreign protein, meat substitution, food adulteration, oilseeds, mass spectrometry, food fraud, food safety, marker peptides

## Abstract

Food fraud is a common issue in the modern food industry. The undeclared use of foreign proteins in meat products is a major concern in this context. Oilseeds are ideal for this purpose due to their high protein content and since huge amounts of oil meal are obtained as a by-product of oil production. Therefore, a UHPLC-MS/MS method was developed for the simultaneous detection of chia, coconut, flaxseed, hemp, peanut, pumpkin, rapeseed, sesame, soy, and sunflower proteins in meat products. Potential tryptic peptide markers were identified by high-resolution mass spectrometry. The final twenty peptide markers selected, which are specific for one of the ten species targeted, were each measured by multiple reaction monitoring. To the best of our knowledge, twelve new heat-stable marker peptides for chia, coconut, flaxseed, pumpkin, rapeseed, sesame and sunflower have not been reported previously. Emulsion-type sausages with 0.01, 0.25, 0.50, 0.75 and 1.00% protein addition by each oilseed species were produced for matrix calibration. No false-positive results were recorded. In the quantification of the ten oilseed species, 466 of 480 measuring data points of the recovery rate in unknown sausages (0.15 and 0.85% protein addition by each oilseed species) were in the accepted range of 80–120%.

## 1. Introduction

The current world population of 7.9 billion is growing and expected to reach 10 billion by 2057 [1]. Consequently, an increasing demand for food, particularly animal protein, is expected and is a serious cause for concern [2]. The main source of animal protein in conventional diets in industrialized countries is meat [3]. It is predicted that global meat production will be 19% higher by the year 2030 compared to the 2015–2017 period [4]. Although meat production is increasing, alternative plant protein sources are needed to cover the demand for protein in nutrition [5]. Meanwhile, there is an increasing interest and demand for reduced-meat or non-meat alternative products [6], and the use of plant protein sources has increased rapidly with the launch of meat alternative products since 2010 [7]. Proteins from oilseeds are a sustainable protein source for the substitution of meat [8].

Until 2017, oilseeds were mainly used for the production of vegetable oils [9,10]. Since 2019, the production rates of oil meals (2019: 403 million tons) have noticeably exceeded the production rates of oils (241 million tons) [11,12]. These oil meals (press cakes) are the remaining residues of oil production (defatted materials) and offer significantly higher protein content than the pure oilseeds (seeds vs. press cakes: peanut: 31 [13] vs. 55% [14]; soy: 41 [13] vs. 49% [15]; rapeseed: 30 [16] vs. 40% [5]; and sunflower: 27 [16] vs. 48% [5]). Adding oilseed meals to meat products can be carried out for technological reasons due to their high protein level [17], but, at the same time, can be a source of nutrients and, therefore, provide pro-health benefits [18]. In addition to the high protein content of ground press cakes, their high global production volumes are reasons for the use of oilseed proteins as meat substitutes and alternatives. The production volumes of the most used oilseeds (in million tons) in 2019 were as follows: soy (336), rapeseed (72), coconut (62), sunflower (56), peanut (50), pumpkin (27), sesame (7), flax seeds (3) and hemp (0.077) [11]. No data are available for chia seeds; however, this oilseed has been recognized as an alternative source of plant protein for human consumption [19]. According to Regulation (EU) 2017/2470, chia is classified as a novel food; however, the incorporation of chia into meat products has not yet been authorized [20]. The most important oilseed species in food production are soy, rapeseed, peanut and sunflower seeds [7], and the use of these oilseed proteins as a protein source in hybrid meat products [21], or for plant-based meat substitutes [22], has already been described in the scientific literature. Within the group of oilseed species, hemp [23], pumpkin seeds [24], sunflower seeds [24] and soy [25,26] are already used in the production of meat analogues and hybrid meat products. However, the addition of foreign protein, such as oilseed proteins, must be stated in the list of ingredients according to the Regulation (EU) No 1169/2011, otherwise it is a fraudulent substitution of meat protein [27].

The extant analytical methods for the detection of oilseed proteins in meat products are mainly focused on peanut, sesame and soy species, representatives of the 14 major food allergens recognized by the European Union (EU) [28]. Apart from immunochemical (e.g., enzyme-linked immunosorbent assays; ELISA) and molecular biological (e.g., polymerase chain reaction; PCR) techniques, high-performance liquid chromatography–tandem mass spectrometric methods (HPLC-MS/MS) are available. In contrast to ELISA and PCR, MS methods enable a high-throughput for the simultaneous detection of many different target proteins in food [29,30]. Mass spectrometric methods for the detection of oilseed species in meat products are limited to soy [29,30], hemp [31], or peanut and soy [32,33]. The *Montowska* working group identified species-specific peptides in oilseed cakes from coconut, evening primrose, hemp, flaxseed, milk thistle, nigella, pumpkin, rapeseed, sesame and sunflower [34]. Furthermore, they proposed peptide markers for the detection of flaxseed, nigella, pumpkin, sesame and sunflower in meat alternative products [31].

The aim of this study was to identify new peptide markers and to develop a UHPLC-MS/MS method for the simultaneous detection of the addition of oilseed proteins from chia (*Salvia hispanica*), coconut (*Cocos nucifera*), flaxseed (*Linum usitatissimum*), hemp (*Cannabis sativa*), peanut (*Arachis hypogaea*), pumpkin (*Cucurbita*), rapeseed (*Brassica napus*), sesame (*Sesamum indicum*), soy (*Glycine max*) and sunflower (*Helianthus annuus*), with a limit of detection (LOD) of 0.01% in meat products. The peptide markers for peanut and soy were adopted from a previous study [32]. New heat-stable peptide markers require to be identified for the eight other oilseed species. The second purpose of application of this method was to quantify the added oilseed protein in the range of 0.01–1.0% (related to the protein content in each oilseed species added to the emulsion-type sausages), which was performed by applying a matrix calibration.

## 2. Materials and Methods

### 2.1. Materials

#### 2.1.1. Chemical Material

The solvents acetone, acetonitrile (ACN) and LC-MS/MS water were purchased from LGC Standards (Wesel, Germany) in Optigrade quality. Ethanol (absolute, p.A.), 2-propanol (LC-MS grade), hydrochloric acid (HCl; p.A.) and formic acid (p.A.) were obtained from Merck (Darmstadt, Germany). Tris(hydroxymethyl)-aminomethane (TRIS; ≥99.3%) was purchased from Carl Roth (Karlsruhe, Germany), trypsin (sequencing grade) from Promega (Madison, WI, USA) and dimethyl sulfoxide (p.A.) from J.T. Baker (Center Valley, PA, USA). Formic acid (for LC-MS) was bought from Honeywell (Charlotte, NC, USA). Iodoacetamide and DL-dithiothreitol (≥98%) were bought from Sigma (St. Louis, MO, USA).

#### 2.1.2. Sample Material

Coconut (*Cocos nucifera*) meal and hemp (*Cannabis sativa*) protein powder were purchased from Govinda Natur GmbH (Neustadt an der Weinstraße, Germany). Peanut (*Arachis hypogaea*) flour and soy (*Glycine max*) protein isolate were obtained from Bulkpowders (Colchester, UK). Pumpkin (*Cucurbita*) protein powder and sunflower (*Helianthus annuus*) protein powder were obtained from Sunflower Family GmbH (Wiggensbach, Germany). Flaxseed (*Linum usitatissimum*) flour was purchased from Rapunzel Naturkost GmbH (Legau, Germany) and rapeseed (*Brassica napus*) flour from Die Ölfreunde (Beuron/Thiergarten, Germany). Sesame (*Sesamum indicum*) protein powder was obtained from Raab Vitalfood GmbH (Rohrbach, Germany), and chia (*Salvia hispanica*) flour from Ölmühle Solingen (Boffzen, Germany).

The materials of each oilseed flour were homogenized and blended to obtain comparable concentrations of oilseed protein from each oilseed species for the sausage of processing series 1 (test sausages) and were blended for the standard and unknown sausages of processing series 2, as shown in Table 1.

### 2.2. Methods

#### 2.2.1. Sample Preparation for Mass Spectrometry

**Figure 1:**
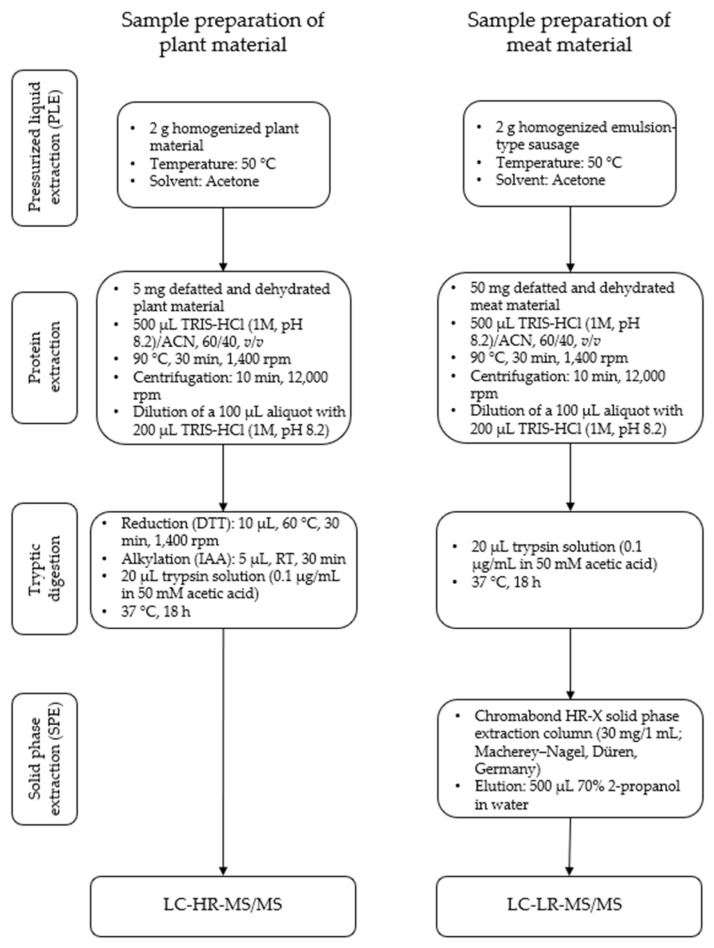
Experimental workflow for the sample preparation of oilseed meals (plant material) for LC-HR-MS/MS and emulsion-type sausages with added oilseed protein (meat material) for LC-LR-MS/MS.

The sample preparation of oilseed flours for high-resolution and homogenized emulsion-type sausages for triple-quadrupole mass spectrometry was performed as described previously [32] (Figure 1). Briefly, the samples were defatted and dehydrated using acetone. The proteins of the defatted samples were extracted with a buffer (TRIS-HCl (1 M, pH 8.2)/ACN, 60/40, *v*/*v*) for 0.5 h at 90 °C. After dilution with TRIS-HCl (1 M, pH 8.2) the plant materials were first reduced and alkylated and afterwards digested using trypsin, whereas the meat materials were digested directly. Afterwards the digested samples were cleaned up using solid phase extraction. The eluates were concentrated in a nitrogen stream and subsequently dissolved in 50 µL of solvent A (see Section 2.2.2).

#### 2.2.2. HPLC-MS/MS-Identification of Peptides for Chia, Coconut, Flaxseed, Hemp, Pumpkin, Rapeseed, Sesame and Sunflower

The experimental procedure comprised the preparation of plant materials as described in Section 2.2.1, liquid chromatography–high-resolution mass spectrometry, and data analysis for peptide identification.

##### Liquid Chromatography–High-Resolution Mass Spectrometry

Liquid chromatography was performed with a Dionex UltiMate 3000 RS HPLC from Thermo Scientific (Waltham, MA, USA) as published previously [32]. The injection volume was 2 μL, the temperature of the column (Nucleosil 100-3 C18 HD column (125 × 2 mm; particle size: 3 μm) from Macherey–Nagel (Düren, Germany)) was set to 40 °C. The mobile phase consisted of solvent A (water with 3% ACN and 0.1% formic acid) and solvent B (water with 90% ACN and 0.1% formic acid). The duration of the LC run with a flow rate of 0.25 mL/min was 52 min. The LC run started with 2% B for 3 min, followed by a gradient to 60% B in 30 min and another gradient to 100% B in 1 min. An isocratic step at 100% B continued for 10 min. After switching to 2% B in 1 min, the column was allowed to equilibrate at 2% B for 7 min.

The data for peptide identification (peak lists of precursor and fragment ions) were obtained by data-dependent high-resolution MS/MS on a maXis ultra-high resolution time-of-flight system (Bruker Daltonik, Bremen, Germany) in positive electrospray ionization (ESI) mode (capillary voltage: 3500 V). The ESI interface setting parameters were as follows: the drying gas temperature was set to 180 °C and the ESI nebulizer gas (N_2_) pressure was 4 bar. The mass range of the LC-MS/MS measurements was *m*/*z* 100–1600 with a spectra scan rate of 2 Hz. Selected precursors analyzed more than twice were actively excluded from analysis for 60 s. The collision energy of the quadrupole ranged between 25 and 50 V [35].

##### Data Analysis for Marker Peptide Identification

The peak lists of the data-dependent MS/MS measurements were analyzed with PEAKS Studio 10.0 (Bioinformatics Solutions, Waterloo, ON, Canada). The following parameters were applied for de novo sequencing: The mass tolerance for the precursor and fragment ions was set to 0.025 Da; the enzyme for digestion was trypsin; no missed cleavages were allowed; cysteine carbamidomethylation was set as a fixed modification. The peptides identified were searched against the NCBI database (version 13 April 2021) with PEAKS Studio, whereas the taxonomy was restricted to Viridiplantae. The raw data lists of peptides identified per oilseed species were imported into JMP 16.1.0 (SAS, Heidelberg, Germany). The peptides were additionally checked for potential homologies in other species using the online search tool of the NCBI database (Protein BLAST; accession date: 24 March 2022). The parameters for database search were as follows: query cover = 100%, percent identity = 100%; with no restriction of the taxonomy. The mass spectrometry proteomics data have been deposited at the ProteomeXchange Consortium via the PRIDE [36] partner repository with the dataset identifier PXD035260.

#### 2.2.3. Synthesis of Peptides

The peptide candidate markers (see Table S1) were synthesized and purified as described previously [37]. The identities of the peptides purified were verified as reported previously [32]. Furthermore, the synthesized peptides were used to select the five most intensive, theoretically explainable, mass transitions for each peptide marker and to optimize their MS/MS parameters (declustering potential (DP), collision energy (CE), and cell exit potential (CXP)) at the AB Sciex QTrap 5500 (Darmstadt, Germany) using syringe pump injection.

#### 2.2.4. UHPLC-MS/MS-Detection of Marker Peptides for the Ten Oilseed Species in Emulsion-Type Sausages

The analytical method comprised the preparation of meat materials as described in Section 2.2.1 and liquid chromatography–triple quadrupole mass spectrometry. 

##### Liquid Chromatography–Triple Quadrupole Mass Spectrometry

Peptide separation (Dionex UltiMate 3000 RS HPLC from Thermo Scientific (Waltham, MA, USA)) and detection (Sciex QTrap 5500, Darmstadt, Germany) was performed as published previously [32]. The mobile phase was described in Section 2.2.2. The temperature of the column (Nucleodur C18 Gravity-SB column; 50 × 2 mm; particle size 1.8 μm) from Macherey–Nagel (Düren, Germany)) was set to 50 °C. The LC run (injection volume 2 µL; flow rate 0.7 mL/min; total time: 15.5 min) started with 2% B and a linear gradient to 30% B in 9.9 min. After switching to 100% B in 0.1 min, an isocratic step continued for 3.5 min at 100% B. After switching to 2% B, the column was allowed to equilibrate for 2.0 min. Peptide detection was carried out in the positive ESI mode. The source temperature was set to 550 °C, ion spray voltage to 3.7 kV, curtain gas flow to 35, and entrance potential to 10 V. Details of the scheduled MRM method are shown in Table 2. The processing of the mass spectrometric data was performed with Analyst 1.7.1 (Sciex, Darmstadt, Germany).

#### 2.2.5. Statistical Analysis

Calculations were performed with JMP (Version 16.1.0; SAS, Heidelberg, Germany). With respect to the sample preparation of each batch of processing series 2, one sample from each of two different cans was defatted and, subsequently, the two defatted materials were each prepared in triplicate to obtain six digested samples for each batch. Each digested sample was measured twice to obtain twelve measuring data points for each batch. The digested samples were measured in three independent measuring sequences. The unknown sausages (U1–U3), although produced with defined concentrations (Table 1), were treated as samples with unknown concentrations. The concentrations of the unknown sausages for each oilseed species were calculated using a matrix calibration derived from the standard sausages (S1–S5; N = 6) of processing series 2. The results of the comparison of the defined and calculated concentrations are represented by recovery rates (N = 12). Standard boxplots were used to visualize these recovery rates. The boxplots depict the median as the central line and the quantiles as boxes. The upper and lower ends of the vertical lines extend to 1.5 times the interquartile distance at most. Outliers are displayed as dots.

## 3. Results and Discussion

### 3.1. Determination of Suitable Marker Peptides for Chia, Coconut, Flaxseed, Hemp, Pumpkin, Rapeseed, Sesame and Sunflower in Plant Material

**Figure 2:**
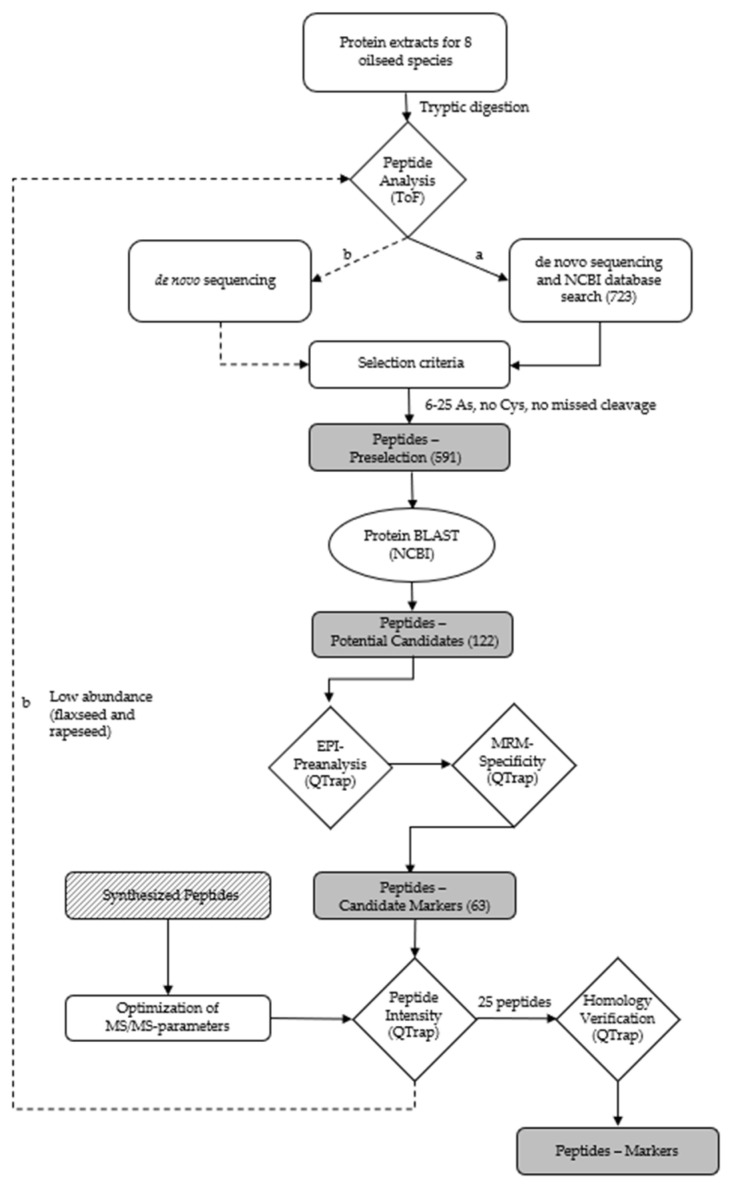
Workflow for the identification of (a) peptides for chia, coconut, flaxseed, hemp, pumpkin, rapeseed, sesame and sunflower via database search, and (b) de novo peptides for flaxseed and rapeseed due to the low abundance of peptides identified by (a).

The workflow for the identification of suitable marker peptides for chia, coconut, flaxseed, pumpkin, rapeseed, sesame and sunflower is shown in Figure 2.

Some of the previous studies that addressed the extraction of oilseed proteins from meat products used an ammonium bicarbonate buffer (100 mM, pH 7.8) at RT to extract either hemp protein [31] from meatballs or soy protein [44,45,49] from poultry products. Others used a TRIS-HCl (1 M, pH 8.2) buffer to extract soy protein at 70 °C [46] or peanut and soy protein at 90 °C [32] from emulsion-type sausages. Furthermore, it was shown that higher extraction temperatures significantly increased the extraction yield of peanut and soy proteins in meat products [32,46]. The TRIS-HCl buffer was preferred due to the high extraction temperatures required for an efficient oilseed protein extraction and the lack of heat stability of ammonium bicarbonate. Furthermore, previous investigations revealed that the addition of ACN to the buffer (TRIS-HCl (1 M, pH 8.2)/ACN, 60:40, *v*/*v*; TA-60/40) increased the extraction yield of peanut and soy proteins in emulsion-type sausages, compared to the addition of 2-propanol or ethanol to the extraction buffer [32].

Consequently, the extraction of the oilseed proteins was performed applying the buffer TA-60/40 for the identification of peptides specific for chia, coconut, flaxseed, hemp, pumpkin, rapeseed, sesame and sunflower by high-resolution mass spectrometry. The specific peptide markers for the oilseed species peanut and soy were adopted from a previous study [32]. The number of accessions (NCBI) for the target proteins of the different oilseed species were 32 for chia, 6 for coconut, 6 for flaxseed, 16 for hemp, 16 for pumpkin, 78 for rapeseed, 11 for sesame and 78 for sunflower. A total of 723 different peptides (chia: 85; coconut: 57; flaxseed: 54; hemp: 75; pumpkin: 137; rapeseed: 105; sesame: 72 and sunflower: 148) were obtained by means of de novo sequencing and a subsequent search of the NCBI database. The peptides had a length of 6–46 amino acids, a mass range of *m*/*z* 403–1328 and an ion charge of 2–5 (Figure 2). Ten peptides were eliminated due to their presence in two oilseed species. A preselection was generated from these 723 peptides by applying the following criteria: a length of 6–25 amino acids, no cysteine and no missed cleavage sites for trypsin [50]. The resulting 591 preselected peptides were searched against the NCBI database using the online BLAST algorithm (https://blast.ncbi.nlm.nih.gov/BLAST.cgi; accessed on 4 November 2021). The peptides had to have entries for only one of the oilseed species analyzed and no entries for other relevant matrices, such as meat species or spices. Due to the high degree of relatedness of Brassicaceae, a family that includes a variety of different species, such as mustard (*Sinapis alba*, *Brassica juncea*, and *Brassica nigra*), various types of cabbage (*Brassica oleracea*) and turnips (*Brassica rapa*), all potential marker peptides for rapeseed showed homologies to different cabbages and turnips which were tolerated because they are not relevant ingredients in meat products. By contrast, peptides showing homologies in mustard were excluded, because mustard is a common spice in meat products. The remaining potential peptide markers (122) for chia (20), coconut (8), flaxseed (15), hemp (8), pumpkin (13), rapeseed (23), sesame (8) and sunflower (27) were verified by an enhanced product ion (EPI) scan measuring the tryptic digests of the oilseed flours. The five most intense fragment ions with *m*/*z* > 250 of each peptide determined by the EPI measurement were used as mass transitions, applying an MRM method with non-optimized MS/MS parameters. The most intense peptides (63) for chia (7), coconut (6), flaxseed (8), hemp (8), pumpkin (7), rapeseed (12), sesame (8) and sunflower (7), according to the MRM measurements, were selected as candidate peptide markers and synthesized (Table S1). A total of 15 out of these 63 candidate peptide markers were identified previously in oilseed cakes (flaxseed: 3, hemp: 2, pumpkin: 2, rapeseed: 5, sesame: 1, and sunflower: 2) [34]. Furthermore, the two mentioned hemp peptides identified in hemp cake and three additional hemp peptides were detected in meat balls [31]. For sesame, three further peptides were detected in bakery products [43,51]. The synthesized peptides were used to select the five most abundant theoretically explainable mass transitions (*m*/*z* > 250 Da) and for the optimization of the MS/MS-parameters of each peptide.

**Figure 3:**
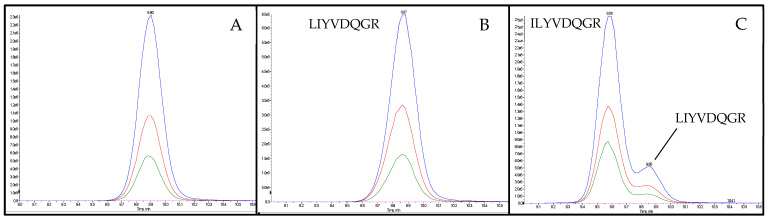
Chromatograms of the peptide marker flaxseed 2 (LIYVDQGR) in tryptic digests of test sausage T5 (Table 1) applying a flat LC-gradient. (**A**) unspiked; (**B**) spiked with synthesized peptide LIYVDQGR; (**C**) spiked with synthesized peptide ILYVDQGR.

Subsequent to the optimization of the MS/MS-parameters, the intensities of the candidate peptide markers in the meat matrix were checked. Accordingly, a spiked sausage (test sausage T5, Table 1) was analyzed applying an optimized MRM method. The eight selected and synthesized candidate peptide markers for the species of flaxseed known from the NCBI database could not provide the necessary intensity for the detection in meat products with 0.1% flaxseed protein (test sausage T5). Consequently, 52 peptides, unknown to the NCBI database but with an average local confidence (ALC) ≥ 85 obtained during de novo sequencing of the flaxseed sample, were reevaluated (Figure 2b). In the end, the two peptides FF(L/I)AGNPQR (flaxseed 1, ALC: 89) and (L/I)(L/I)YVDQGR (flaxseed 2, ALC: 92) were selected as candidate peptide markers for flaxseed by applying the same criteria as mentioned before. Since a mass spectrometric differentiation of leucine (L) and isoleucine (I) was not possible, the isomeric peptides FFLAGNPQR, FFIAGNPQR, LLYVDQGR, LIYVDQGR, ILYVDQGR and IIYVDQGR were synthesized for the verification of the peptide marker’s correct identities by spiking them into a tryptic digest of an emulsion-type sausage with added flaxseed flour (test sausage T5). The isomeric peptide of flaxseed 1, FFIAGNPQR, could be distinguished from the final selected peptide marker FFLAGNPQR due to different retention times (t_R_s). Furthermore, only FFLAGNPQR coeluted with the endogenous peptide, confirming its identity as flaxseed 1. Both alternative sequences of flaxseed 2, LLYVDQGR and IIYVDQGR, could be excluded as the correct identity of the peptide marker due to different retention times (t_R_s) when applying the final MS/MS method. The isomeric peptides ILYVDQGR and LIYVDQGR both coeluted with the endogenous peptide applying the final LC-MS/MS method. However, the identity of flaxseed 2 could be determined as LIYVDQGR by a chromatographic separation applying a flat LC-gradient (Figure 3). The two peptide markers FFLAGNPQR and LIYVDQGR, and their isomers, showed no homologies (except for bacteria). The identical process of de novo sequencing was also performed for rapeseed; however, only one out of 20 peptides (ALC ≥ 85) identified (VQGPFSVLRPPLR) passed the selection criteria (6–25 amino acids, no cysteine, and no missed cleavage) and was synthesized. Unfortunately, this peptide did not survive the step of checking the intensity in the meat matrix.

**Figure 4:**
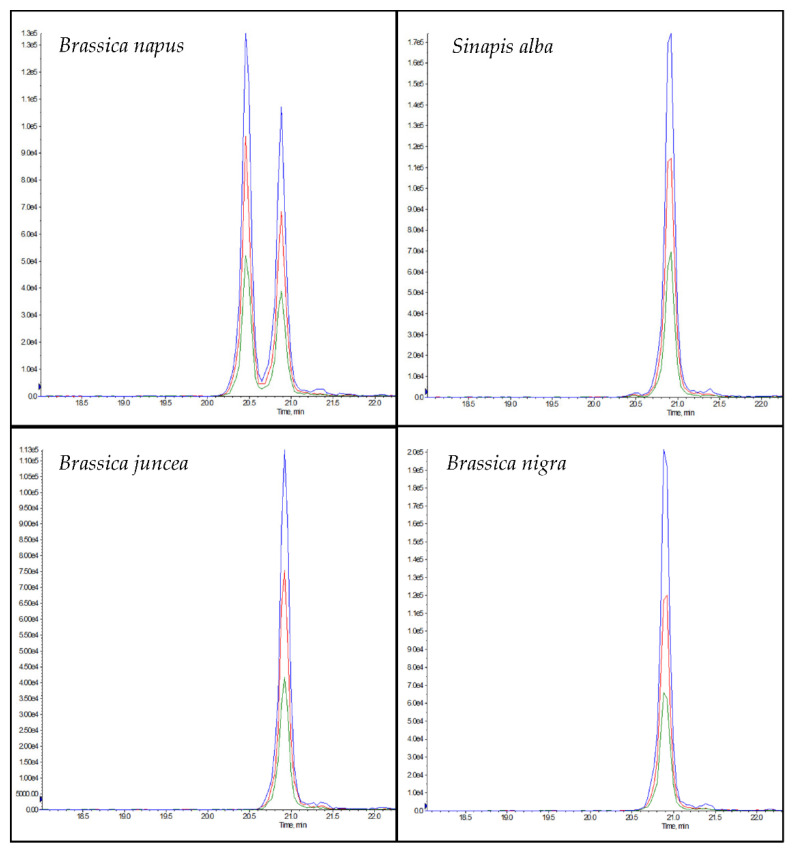
Chromatograms of the peptides NLRPFLIAGNNPQGQQWLQGR (rapeseed 1, RT: 22.5 min) and NLRPFLLAGNNPQGQQWLQGR (isomer to rapeseed 1, t_R_: 22.9 min) in the different pure plant flours of rapeseed (*Brassica napus*), white mustard (*Sinapis alba*), brown mustard (*Brassica juncea*), and black mustard (*Brassica nigra*).

The chromatogram of NLRPFLIAGNNPQGQQWLQGR (rapeseed 1) in rapeseed samples showed a double peak in the chromatogram (Figure 4), which can be explained by the isomeric peptide NLRPFLLAGNNPQGQQWLQGR, which could be differentiated from the peptide marker by a later retention time (Figure 4). According to the NCBI database, the isomeric peptide has homologies in the mustard species *Brassica carinata* and *Sinapis alba* (Table S2). In addition, the isomeric peptide showed homologies to the mustard species *Brassica juncea*, *Brassica nigra* and *Sinapis alba* when tryptic digests of the different mustard flours were analyzed (Figure 4). However, only the posterior peak belonging to NLRPFLLAGNNPQGQQWLQGR was observed, and not the anterior peak belonging to NLRPFLIAGNNPQGQQWLQGR. Although rapeseed 1 contained a high number of asparagine (N) and glutamine (Q) residues, in tests performed with the synthesized peptide, no deamidation products were detected during protein extraction despite the high temperature (90 °C) and a pH > 7.

In addition to the comparison with the NCBI database (Table S2), the uniqueness of the remaining 25 peptide candidate markers for chia (3), coconut (3), flaxseed (2), hemp (3), pumpkin (3) rapeseed (4), sesame (4) and sunflower (3) was confirmed experimentally by analyzing the ten oilseed flours and an emulsion-type sausage without the addition of any type of oilseed (blank value) using the optimized MRM method. The uniqueness of the peptides was then further verified by analyzing a total of 121 possible ingredients of meat products and nine commercial spice mixtures divided into eighteen groups (Table S3) using the optimized MRM method. Casein powder was added to each of the eighteen groups as a control for the successful tryptic digestion and the correct performance of the LC-MS/MS measurement to eliminate false-negative results. Therefore, the two casein peptides FFVAPFPEVFGK [30] and YLGYLEQLLR [30] were integrated into the MRM method (Table S5 and Table 2).

None of the candidate peptide markers of chia, coconut, flaxseed, hemp, pumpkin, sesame, or sunflower were detected in the meat matrix, the 121 ingredients or the spice mixtures. Three of the candidate peptide markers for rapeseed—AHEAHDTSLTTETR LTFVVHGHALMGK and QQQGQQGQQLQQVISR—were detected in group 9 (“Others”, Table S3) and in group 18 (“Commercial spice mixtures”, Table S3). The members of these two groups were analyzed individually to determine the specific ingredients that caused the detection of the three peptide markers. The peptides were detected in the three mustard species *Brassica juncea*, *Brassica nigra* and *Sinapis alba* for group 9, and the peptides were detected again in *Sinapis alba*, which is a common ingredient in curry mixtures [52], for group 18. Only one candidate peptide marker of rapeseed, NLRPFLIAGNNPQGQQWLQGR, was not detected in the mustard species or commercial spices. Although the flaxseed peptide marker FFLAGNPQR has homologies (predicted) according to the BLAST search (Table S2) in potato (group 6: “Roots and Tubers”, Table S3) and tomato (group 7: “Fruit Vegetables”, Table S3), these homologies could not be confirmed experimentally.

It was not possible to identify two unique peptide markers for rapeseed due to the high degree of relatedness of the Brassicaceae, an incomplete coverage by the NCBI database and the insufficient intensities of the peptide candidate markers for rapeseed. In order to be able to exclude the presence of mustard in meat products, a peptide marker specific to mustard, ALPLEVITNAYQISLEEAR, was identified as a peptide marker and can be integrated in the method (Table S4). The peptide is unique to the mustard species *Brassica juncea*, *Brassica nigra* and *Sinapis alba* and shows no homology to rapeseed according to the NCBI database or in experimental verification. Although the peptide marker QQQGQQGQQLQQVISR is considered to be unique to rapeseed according to the NCBI database, experimental tests showed homologies to the mustard species *Brassica juncea*, *Brassica nigra* and *Sinapis alba*. However, the peptide QQQGQQGQQLQQVISR was selected as the second peptide marker for rapeseed due to a higher peak intensity than the two other candidate peptide markers, AHEAHDTSLTTETR and LTFVVHGHALMGK, which have been proposed as peptide markers in the scientific literature [34].

According to the requirements to use two peptide markers (quantifier and qualifier) for the mass spectrometric detection of allergens in foodstuffs [53], two peptides for each oilseed species were finally chosen as peptide markers. A third peptide was determined for each of the species, chia, coconut, hemp, peanut, pumpkin, sesame and soy, as a possible alternative (Table S5). The final peptides selected: GTLDLVSPLR and ILAESFNVDTELAHK for hemp [31], LVFVAQGFGIR for pumpkin [34], AFYLAGGVPR for sesame [43], FPILEHLQLSAER for sunflower [34] and the alternative peptides FYIAGNPHEDFPQSR for hemp, ISTANYHTLPVLR for pumpkin and ISGAQPSLR for sesame have been reported previously.

The identities of the twelve new heat-stable peptide markers ELQVIKPPFR, GPIVIVEK and NTLRPNALSLPNYHPNPR for chia, EVDEVLNAPR, LNALEPTR and GLLLPSMSNAPR for coconut, FFLAGNPQR and LIYVDQGR for flaxseed, VLAEIFNINVETAR for pumpkin, NLRPFLIAGNNPQGQQWLQGR for rapeseed, LVLPEYGR for sesame, and FPILEHLR for sunflower, which, to the best of our knowledge, have not been reported in the scientific literature until now, were confirmed by spiking them into a tryptic digest of an emulsion-type sausage with added oilseed flour (test sausage T5; Table 1). According to the NCBI database, the new peptide markers for chia (GPIVIVEK, ELQVIKPPFR), flaxseed (LIYVDQGR), pumpkin (VLAEIFNINVETAR), rapeseed (NLRPFLIAGNNPQGQQWLQGR) and sunflower (FPILEHLR) showed no food-relevant homologies (Table S2). The alternative peptide marker for chia (NTLRPNALSLPNYHPNPR) is also present in perilla (*Perilla frutescens*). The coconut peptide markers can also be found in date palm (*Phoenix dactylifera*) and/or in oil palm (*Elaeis guineensis*) and should be understood as markers for the members of the palm tree family (*Arecaceae*) mentioned. The peptide marker for flaxseed (FFLAGNPQR) is also present in kiwi fruit (*Actinidia chinensis*), which is used as a meat tenderizer [54]. However, its use is limited to larger meat pieces, and the addition to meat products, such as emulsion-type sausages, is not appropriate. Furthermore, the homologies predicted in different potato and tomato species were not confirmed by the experimental homology tests (Table S3). Despite the peptide marker for sesame (LVLPEYGR) also being present in Atlantic herring (*Clupea harengus*), this homology was not relevant because it was not a tryptic peptide. The twelve new peptide markers mentioned showed no homologies in 121 possible ingredients and ten commercial spice mixtures for meat products (Table S3).

### 3.2. Detection of Oilseed Peptide Markers and Quantification of Protein Addition by Oilseed Proteins in Emulsion-Type Sausages

The production of meat products focused on emulsion-type sausages due to their high homogeneity and the available technological experience with the addition of vegetable protein [35,46]. The emulsion-type sausages of processing series 1 and 2 (Table 1) were produced as full preserves (F-value = 5–6). This type of preservation reflects the most intense thermal processing (core temperature of 121 °C for 5–6 min) commonly used for production of emulsion-type sausages. This procedure should ensure that the oilseed proteins in the sausages were subjected to high temperatures and, consequently, that successful detection of the peptide markers would be an indicator of their heat stability. In order to confirm the heat stability of the peptide markers, one batch of emulsion-type sausages was produced, which was processed as full preserves as well as semi-preserves—the latter reflect the lowest common thermal processing (core temperature of 65–75 °C). The analysis of seven samples for each thermal processing showed that the peak areas of the peptide markers in the full preserves were a mean 22% lower (from 8% for hemp 1 and pumpkin 2 to 45% for peanut 1) compared to semi-preserves. Rapeseed 1 showed an average decrease (20%) despite the high number of N and Q mentioned, and, therefore, a possible deamidation during sausage processing at higher temperature would have occurred only to a minor extent. The mentioned decreases in the peak areas in the samples of the full preserves were not necessarily a result of a lack of heat stability. They might have been caused by an ion suppression by the meat matrix since the peak areas of two myosin peptides included in the method (see below) were, on average, about a factor of seven higher in the full preserves compared to the semi-preserves (N = 7, each).

**Figure 5:**
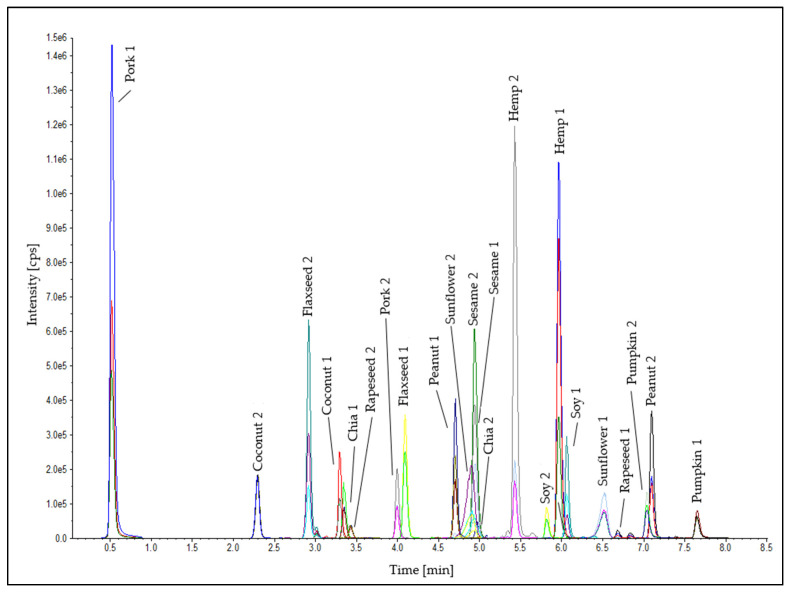
Chromatogram of the peptide markers for the oilseeds and the meat matrix in the test sausage T5 (0.1% oilseed protein, Table 1).

Two peptide markers for each oilseed species were used for the detection in the emulsion-type sausages. Furthermore, two heat-stable peptide markers for the meat matrix (pork 1: SALAHAVQSSR [55,56] and pork 2: DTLVSQLSR [57]) were established in the analytical method (Table S4) as internal control standards to check the successful tryptic digestion and the correct performance of the LC-MS/MS measurement to eliminate false-negative results for the detection of the oilseed species. A chromatogram of the oilseed and meat peptide markers in an emulsion-type sausage (0.1% oilseed protein; test sausage T5; Table 1) is shown in Figure 5. The standard deviations of the retention times of all peptide markers were ≤±0.02 min (Table 2). This stability of the retention times is a meaningful quality feature for the reliable detection of the peptide markers in the method developed. No false-positive results were obtained in either processing series.

All oilseed flours were added at the same protein levels for production of the five batches of processing series 1 (0.0005–0.1% oilseed protein; test sausages T1–T5; Table 1). These test sausages were used for the final selection of the two best-performing peptide markers for each oilseed species and the determination of the LOD for each target. The three most abundant mass transitions of both peptide markers of a given target must have a signal-to-noise ratio (S/N) equal or greater than three, according to the commonly used definition of the LOD for a reliable detection [58]. The LODs were ≤0.0005% for flaxseed, ≤0.001% for hemp, peanut, and sesame, ≤0.005% for chia, coconut, pumpkin, soy, and sunflower, and ≤0.01% for rapeseed, related to the respective oilseed protein content in the emulsion-type sausage (full preserves). The LOD mentioned for hemp (0.002% hemp protein powder) was a factor of about 500 lower than the LOD reported previously for the mass spectrometric detection of hemp cake in meat products of below 1% [31]. In consequence of the differences in the peak areas between the semi-preserves and the full preserves described above, slightly lower LODs were expected for emulsion-type sausages produced as semi-preserves.

Based on the determination of the LODs using the sausages of processing series 1, the lowest protein concentration at which all oilseed species were detectable (0.01% oilseed protein, each) served as the lowest concentration for processing series 2, which was used for quantification. It was assumed that a quantification at concentrations lower than 0.01% was not useful since these low concentrations are not economically viable for manufacturers of meat products. A successful quantification of rapeseed revealing the highest LOD (0.01%) was expected since the S/N ratios of both rapeseed peptides at the concentration of 0.01%were ≥12 and therefore above the limit of quantification (LOQ). The oilseed flours used for production of processing series 2 were divided into two groups (five species each) to obtain comparable oilseed protein contents of the added meals in both groups (mean protein content in group A [chia, flaxseed, hemp, pumpkin and sesame]: 44.9%, and in group B [coconut, peanut, rapeseed, soy, and sunflower]: 45.5%). This division was carried out in order to have the opportunity to introduce higher protein concentrations up to 1% for each oilseed species into the sausages while retaining the characteristic consistency of emulsion-type sausages. Standard and unknown sausages were produced for the quantification of each oilseed species in processing series 2. The standard sausages (S1a/b–S5 a/b: 0.01, 0.25, 0.5, 0.75 and 1.0%) were produced in such a manner that each batch included the five concentration levels represented, alternating by another oilseed species (Table 1). The unknown sausages (U1 a/b–U3 a/b: 0, 0.15 and 0.85%) were produced under the same conditions as the standard sausages, whereby 1–2 oilseed species were missing in each batch (Table 1).

Three criteria were considered for the standard sausages to check the suitability of the peptide markers for the quantification and to decide which peptide marker should be used as a quantifier: (a) the mean coefficients of determination (R^2^) of the regression between the concentration of oilseed protein and the peak area of a peptide marker; (b) the relative standard deviations (RSD) of the ratio of the lowest to highest intensive mass transition peak area as an indicator of the precision of the peak integration of a peptide; and (c) the RSD of the repeatability of the peak area at varying concentrations of oilseed protein (Table 3). Regarding criterion (a), all peptide markers met the requirement (R^2^ ≥ 0.9). Furthermore, all peptide markers, with the exception of rapeseed 2, fulfilled criterion (b) at all concentration levels (RSD < 20%). Concerning criterion (c), the peptide markers for pumpkin 2 and sunflower 2 showed higher RSDs (>20%) at several concentration levels. Furthermore, regarding the peptide markers of flaxseed 2 and rapeseed 2, higher values for criterion (c) (RSD > 20%), at the concentration level of 0.25%, and at the two highest concentration levels (0.75 and 1.0%), were obtained for the peptide marker pumpkin 1. According to the results of the performance criteria mentioned the quantifiers (peptide 1) and qualifiers (peptide 2) of each oilseed species were determined as shown in Table 3.

**Figure 6:**
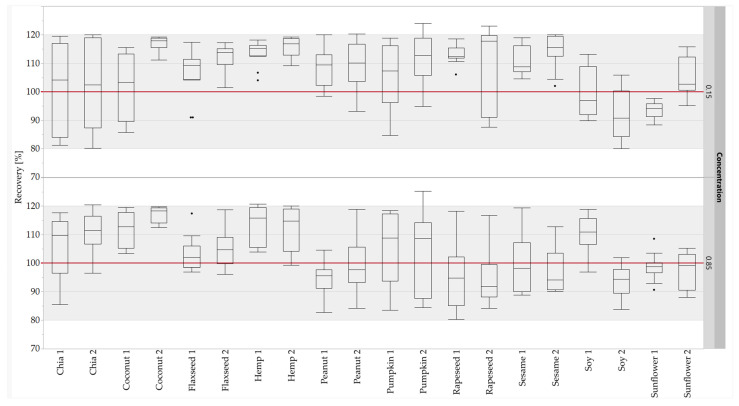
Recovery rates of the oilseed peptides in emulsion-type sausages (0.15 and 0.85% protein) quantified with standard sausages (0.01–1.0% oilseed protein content). Both concentration levels (0.15 and 0.85%) were measured in duplicate from two different sausage samples and three independent sample preparations: box plots are from twelve measurements. The gray areas represent the accepted range of 80–120%.

The calibration curves (five-point calibration) of the standard sausages were used to quantify the oilseed protein concentrations in the unknown sausages. The concentrations were calculated for the quantifier and the qualifier of each oilseed species. The concentrations calculated were compared with the corresponding theoretical concentrations to obtain the recovery rates. About 97% of the measuring data (466 out of 480) for the recovery rates of the peptide markers were within the accepted range of 80–120% [59] (Figure 6). No recovery rates below 80% were observed at either concentration level. Furthermore, the 75-percentiles of all recovery rates were ≤120%. All results for the recovery rates for both concentration levels (0.15 and 0.85%) were within the accepted range, with the exception of hemp 1 (0.85%), pumpkin 2 (0.15 and 0.85%) and rapeseed 2 (0.15%). Based on the results of the quantification, all peptide markers were suitable for the quantification, with the exception of pumpkin 2 and rapeseed 2. Only the quantifier was suitable for the quantification for pumpkin and rapeseed, because the high RSDs of the repeatability of pumpkin 2 were confirmed by the results for the recovery rates, and rapeseed 2 was detected in mustard species. Despite the high RSDs of the repeatability of sunflower 2, the peptide was suitable for quantification due to the sufficient results of the recovery rates. The quantification results for the peptide markers for peanut and soy were comparable with the results obtained for sausages with added legume proteins [32], demonstrating the robustness of these peptide markers against different matrix components.

## 4. Conclusions

A UHPLC-MS/MS method for the simultaneous detection of proteins from ten oilseed species in thermally treated meat products was developed. After a comprehensive selection procedure, the most suitable marker peptides for the detection of low amounts of oilseed proteins in meat products were selected. In this context, twelve new heat-stable and specific peptide markers for the species, chia, coconut, flaxseed, pumpkin, rapeseed, sesame, and sunflower, were identified. It was possible to avoid the time-consuming preparative steps of reduction and alkylation by the exclusion of peptides containing cysteine, as proposed in the scientific literature [31,34,60]. The method developed allows a fast multiplex detection in less than 16 min measuring time (not including preparative steps), outcompeting the speed and the multiplex capability of methods based on ELISA or PCR. Furthermore, it is possible to quantify the added amounts of the oilseed species mentioned in meat products using a matrix calibration in the range of 0.01–1% oilseed protein.

The objective of further studies will be to extend the mass spectrometric method developed for oilseed protein with legume proteins [32] and further relevant plant and animal protein sources for the detection of foreign protein in meat products in a multi-method approach allowing the screening of more than 20 different protein sources. In this context, including alternative protein sources, such as mushrooms [61] or cottonseed [62] should also be considered, which could be of great importance due to high production rates and high protein contents. A requirement for the use of cottonseed protein in food is the reliable detoxification of the inherent gossypol due to its toxicity to humans [62].

## Figures and Tables

**Table 1:** Batches and formulations of sausages with different concentrations of oilseed flours for processing series 1 (test sausages T1–T5: 0.0005, 0.001, 0.005, 0.01 and 0.1% oilseed protein, each) and processing series 2 (standard: S1a–S5a and S1b–S5b; unknown: U1a–U3a andU1b–U3b). Control batches were produced separately for processing series 1 and 2.

	Control	Processing Series 1					Processing Series 2															
		Test Sausages					Standard Sausages								Unknown Sausages							
		T1	T2	T3	T4	T5	S1a	S1b	S2a	S2b	S3a	S3b	S4a	S4b	S5a	S5b	U1a	U1b	U2a	U2b	U3a	U3b
**Formulations (%)**																						
Pork	50	49.987	49.97	49.9	49.7	47.3	45.2	44.9	44.4	43.7	43.6	41.5	43.6	42.0	43.9	41.2	47.4	46.5	45.7	45.4	45.3	43.4
Back fat	24	24	24	24	24	24	24	24	24	24	24	24	24	24	24	24	24	24	24	24	24	24
Curing salt	1.8	1.8	1.8	1.8	1.8	1.8	1.8	1.8	1.8	1.8	1.8	1.8	1.8	1.8	1.8	1.8	1.8	1.8	1.8	1.8	1.8	1.8
Phosphate	0.2	0.2	0.2	0.2	0.2	0.2	0.2	0.2	0.2	0.2	0.2	0.2	0.2	0.2	0.2	0.2	0.2	0.2	0.2	0.2	0.2	0.2
Ice	24	24	24	24	24	24	24	24	24	24	24	24	24	24	24	24	24	24	24	24	24	24
Flour mixture	-	0.013	0.03	0.1	0.3	2.7	4.8	5.1	5.6	6.3	6.4	8.5	6.4	8.0	6.1	8.8	2.6	3.5	4.3	4.6	4.7	6.6
**Oilseed Flour (%)**																						
Chia	-	0.0017	0.0034	0.017	0.034	0.34	0.03	-	0.85	-	1.69	-	2.54	-	3.39	-	-	-	0.50	-	2.88	-
Coconut	-	0.003	0.006	0.03	0.06	0.6	-	0.06	-	1.49	-	2.98	-	4.46	-	5.95	-	-	-	0.89	-	5.06
Flaxseed	-	0.0015	0.003	0.015	0.03	0.3	0.64	-	1.28	-	1.92	-	2.56	-	0.03	-	0.38	-	2.18	-	-	-
Hemp	-	0.0012	0.0024	0.012	0.024	0.24	1.18	-	1.78	-	2.37	-	0.02	-	0.59	-	2.01	-	-	-	0.36	-
Peanut	-	0.0012	0.0023	0.012	0.023	0.23	-	0.56	-	1.13	-	1.69	-	2.25	-	0.02	-	0.34	-	1.91	-	-
Pumpkin	-	0.0008	0.0017	0.008	0.017	0.17	1.27	-	1.69	-	0.02	-	0.42	-	0.84	-	-	-	0.25	-	1.44	-
Rapeseed	-	0.0017	0.0034	0.017	0.034	0.34	-	1.68	-	2.52	-	3.36	-	0.03	-	0.84	-	2.85	-	-	-	0.50
Sesame	-	0.0009	0.0019	0.009	0.019	0.19	1.65	-	0.02	-	0.41	-	0.83	-	1.24	-	0.25	-	1.40	-	-	-
Soy	-	0.0006	0.0012	0.006	0.012	0.12	-	0.89	-	1.18	-	0.01	-	0.30	-	0.59	-	-	-	0.18	-	1.01
Sunflower	-	0.0005	0.001	0.005	0.01	0.1	-	1.92	-	0.02	-	0.48	-	0.96	-	1.44	-	0.29	-	1.63	-	-
**Oilseed Protein (%)**																						
Chia	-	0.0005	0.001	0.005	0.01	0.1	0.01	-	0.25	-	0.50	-	0.75	-	1.0	-	-	-	0.15	-	0.85	-
Coconut	-	0.0005	0.001	0.005	0.01	0.1	-	0.01	-	0.25	-	0.50	-	0.75	-	1.0	-	-	-	0.15	-	0.85
Flaxseed	-	0.0005	0.001	0.005	0.01	0.1	0.25	-	0.50	-	0.75	-	1.0	-	0.01	-	0.15	-	0.85	-	-	-
Hemp	-	0.0005	0.001	0.005	0.01	0.1	0.50	-	0.75	-	1.0	-	0.01	-	0.25	-	0.85	-	-	-	0.15	-
Peanut	-	0.0005	0.001	0.005	0.01	0.1	-	0.25	-	0.50	-	0.75	-	1.0	-	0.01	-	0.15	-	0.85	-	-
Pumpkin	-	0.0005	0.001	0.005	0.01	0.1	0.75	-	1.0	-	0.01	-	0.25	-	0.50	-	-	-	0.15	-	0.85	-
Rapeseed	-	0.0005	0.001	0.005	0.01	0.1	-	0.50	-	0.75	-	1.0	-	0.01	-	0.25	-	0.85	-	-	-	0.15
Sesame	-	0.0005	0.001	0.005	0.01	0.1	1.0	-	0.01	-	0.25	-	0.50	-	0.75	-	0.15	-	0.85	-	-	-
Soy	-	0.0005	0.001	0.005	0.01	0.1	-	0.75	-	1.0	-	0.01	-	0.25	-	0.50	-	-	-	0.15	-	0.85
Sunflower		0.0005	0.001	0.005	0.01	0.1	-	1.0	-	0.01	-	0.25	-	0.50	-	0.75	-	0.15	-	0.85	-	-

**Table 2:** Parameters of the scheduled MRM method (MRM detection window was set to 40s; CE = collision energy; CXP = cell exit potential; DP = declustering potential). The product ions are listed in decreasing intensity. Peptide 1 represents the quantifier, peptide 2 the qualifier (Section 3.2). * Homologies to mustard (*Brassica juncea*, *Brassica nigra*, and *Sinapis alba*) were detected experimentally.

	Peptide Marker	t_R_ [Min]	DP [V]	*m*/*z* (Charge State)	Product Ions	CE [V]	CXP [V]
Chia 1	GPIVIVEK	3.39 ± 0.01	41	427.8 (+2)	587.4 (y5), 488.3 (y4), 700.5 (y6)	19/19/17	42/22/36
Chia 2	ELQVIKPPFR	4.99 ± 0.01	116	409.6 (+3)	322.7 (y5+2), 516.3 (y4), 428.8 (y7^2+^)	13/17/15	24/28/20
Coconut 1	EVDEVLNAPR	3.31 ± 0.01	100	571.3 (+2)	457.3 (y4), 913.5 (y8), 343.2 (y3)	25/23/23	26/50/24
Coconut 2	LNALEPTR	2.31 ± 0.01	71	457.3 (+2)	502.3 (y4), 373.2 (y3), 686.4 (y6)	21/21/17	32/18/32
Flaxseed 1	FFLAGNPQR	4.13 ± 0.01	86	525.3 (+2)	746.4 (y6), 409.2 (y7^2+^), 618.4 (y5)	29/27/27	50/28/28
Flaxseed 2	LLYVDQGR	2.93 ± 0.01	91	482.3 (+2)	737.4 (y6), 360.2 (y3), 574.3 (y5)	21/33/25	42/20/32
Hemp 1	GTLDLVSPLR [31,34]	5.98 ± 0.02	90	535.8 (+2)	472.3 (y4), 571:4 (y5), 799.5 (y7)	25/23/29	22/18/46
Hemp 2	ILAESFNVDTELAHK [31]	5.45 ± 0.01	100	562.9 (+3)	730.9 (y13^2+^), 813.4 (y7), 787.4 (y14^2+^)	19/25/21	36/40/54
Peanut 1	FNLAGNHEQEFLR [38,39,40,41]	4.75 ± 0.01	61	525.6 (+3)	262.1 (b2), 657.3 (y11^2+^), 600.8 (y10^2+^)	23/23/23	14/40/16
Peanut 2	WLGLSAEYGNLYR [38,42]	7.14 ± 0.01	16	771.4 (+2)	272.2 (a2), 300.2 (b2),357.2 (b3)	39/35/39	14/18/18
Pumpkin 1	VLAEIFNINVETAR	7.69 ± 0.01	95	794.9 (+2)	413.2 (b3), 689.4 (y6) 1063.6 (y9)	35/37/35	20/38/46
Pumpkin 2	LVFVAQGFGIR [34]	7.08 ± 0.01	75	603.9 (+2)	748.4 (y7), 497.8 (y9^2+^), 360.2 (b3)	29/23/29	48/24/20
Rapeseed 1	NLRPFLIAGNNPQGQQWLQGR	6.72 ± 0.01	171	803.1 (+2)	599.3 (y10^2+^), 360.2 (y3), 473.3 (y4)	33/33/37	36/18/30
Rapeseed 2 *	QQQGQQGQQLQQVISR	3.45 ± 0.01	116	618.7 (+2)	730.4 (y6), 602.3 (y5), 375.2 (y3)	29/25/27	36/32/20
Sesame 1	AFYLAGGVPR [43]	4.99 ± 0.01	91	525.8 (+2)	485.3 (y5), 382.2 (b3), 566.3 (b5)	23/21/19	28/24/30
Sesame 2	LVLPEYGR	4.88 ± 0.01	71	473.8 (+2)	621.3 (y5), 367.7 (y6^2+^), 326.2 (b3)	21/19/17	28/18/20
Sunflower 1	FPILEHLQLSAER [34]	6.55 ± 0.02	100	518.3 (+3)	469.3 (y12^3+^), 703.4 (y12^2+^), 654.9 (y11^2+^)	25/25/23	24/30/32
Sunflower 2	FPILEHLR	4.95 ± 0.01	76	342.2 (+3)	439.3 (y7^2+^), 667.4 (y5), 390.7 (y6^2+^)	15/19/15	20/38/16
Soy 1	FYLAGNQEQEFLK [44,45,46,47]	6.10 ± 0.02	36	793.9 (+2)	311.1 (b2), 424.2 (b3); 638.7 (y11^2+^)	41/35/33	18/26/38
Soy 2	EAFGVNMQIVR [41,46,47,48]	5.85 ± 0.02	61	632.3 (+2)	760.4 (y6), 387.3 (y3), 532.3 (y9^2+^)	29/29/27	38/22/34

**Table 3:** Limits of detection (LOD), mean coefficients of determination (R^2^) of the regression between the concentration of oilseed protein and the peak area of the peptide markers (a) (N = 6, each), and relative standard deviations (RSD) of the ratio of the lowest to the highest intensive mass transition each (b), and the repeatability of the peak area at varying concentrations of oilseed protein in the standard sausages of processing series 2 (c) as criteria for the suitability of the peptide markers for the quantification; gray marking = RSD ≥ 20%.

Peptide Marker			Concentration of Oilseed Protein [%]									
			0.01	0.25	0.5	0.75	1.0	0.01	0.25	0.5	0.75	1.0
	LOD [%]	R^2^ (a)	RSD [%] of the Mass Transition Ratio (b)					RSD [%] of the Repeatability (c)				
Chia 1	0.005	0.987	4	2	2	3	3	16	9	8	9	11
Chia 2		0.978	13	3	3	3	2	16	11	13	14	11
Coconut 1	0.005	0.993	2	6	14	7	6	8	12	9	10	9
Coconut 2		0.921	2	1	10	1	11	9	10	11	10	6
Flaxseed 1	0.0005	0.994	3	2	1	2	1	6	4	4	7	6
Flaxseed 2		0.980	2	14	7	1	1	5	44	5	6	5
Hemp 1	0.001	0.966	2	2	2	3	4	13	13	10	9	10
Hemp 2		0.958	2	3	5	5	5	14	14	10	9	12
Peanut 1	0.001	0.988	7	3	4	8	9	12	9	10	9	7
Peanut 2		0.991	7	15	5	5	8	14	18	11	13	13
Pumpkin 1	0.005	0.983	10	2	2	2	3	18	15	21	25	22
Pumpkin 2		0.914	4	3	2	2	2	35	34	45	46	53
Rapeseed 1	0.01	0.976	10	14	12	5	11	9	13	13	7	15
Rapeseed 2		0.957	10	9	10	5	21	17	22	16	16	16
Sesame 1	0.001	0.995	2	2	1	2	1	12	16	9	11	9
Sesame 2		0.990	2	1	2	1	1	10	4	4	5	3
Soy 1	0.005	0.993	3	3	4	2	2	5	10	14	3	7
Soy 2		0.986	5	13	5	13	15	15	20	16	5	14
Sunflower 1	0.005	0.994	4	2	2	8	10	13	10	12	11	15
Sunflower 2		0.976	4	2	13	4	5	21	21	37	22	19

## Data Availability

Data is contained within the article or the Supplementary Material.

## Supplementary Materials

The following supporting information can be downloaded at: https://www.mdpi.com/article/10.3390/foods11142155/s1, Table S1: Synthesized peptide candidate markers for the oilseed species chia, coconut, flaxseed, hemp, pumpkin, rapeseed, sesame, and sunflower and corresponding target proteins (peptide markers in bold were selected for the final method; 1 = quantifier, 2 = qualifier, 3 = alternative); Table S2: Peptide markers for chia, coconut, flaxseed, hemp, pumpkin, rapeseed, sesame and sunflower and their homologies (NCBI online search tool BLAST, parameters for database search: query cover = 100%, percent identity = 100%; without bacteria); Peptide 1 = quantifier, Peptide 2 = qualifier, Peptide 3 = alternative peptide marker; * Target proteins refer to *Salvia splendens*; ** predicted homologies according to the NCBI database, which were not confirmed experimentally. Target proteins of pumpkin refer to *Cucurbita maxima*; Table S3: Groups of possible ingredients for the production of sausages and commercial spice mixtures, which were tested regarding cross-reactivity with the oilseed marker peptides analyzed; Table S4: Parameters of scheduled MRM method for peptide markers of casein and pork (MRM detection window 40 s; CE = collision energy; CXP = cell exit potential; DP = declustering potential). The product ions are listed in decreasing intensity; Table S5: Parameters of the scheduled MRM method for alternative peptide markers for the oilseed species (MRM detection window 40 s; CE = collision energy; CXP = cell exit potential; DP = declustering potential). The product ions are listed in decreasing intensity.
