# Supplementary material for: Simultaneous Mass Spectrometric Detection of Proteins of Ten Oilseed Species in Meat Products
Source: Foods. 2022 Jul 20;11(14):2155. doi: 10.3390/foods11142155 (PMC9323756; doi:10.3390/foods11142155)
Supplement: Supplementary file 1 [file foods-11-02155-s001.zip › foods-1793532-supplementary.pdf]

Supplementary Table S1. Synthesized peptide candidate markers (including *de novo* peptides) for the oilseed species chia, coconut, flaxseed, hemp, pumpkin, rapeseed, sesame, and sunflower and corresponding target proteins (peptide markers in bold were selected for the final method; 1 = quantifier, 2 = qualifier, 3 = alternative).

Oilseeds species	Peptide sequence	Target Protein (NCBI Accession)	References
Chia	**ELQVIKPPFR (2)**	11S Globulin-like (P_042056128)	
	GLSNEILAEAFDVDEETAR	11S Globulin-like (XP_042050047)	
	**GPIVIVEK (1)**	11S Globulin-like (P_042056128)	
	GQQHEMGNIFR	11S Globulin-like (XP_042050047.1)	
	NLDHPTSADLYNPR	Legumin B-like (XP_042059362)	
	NTLRPNALSLPNYHPNPR (3)	11S Globulin Seed Storage Protein 2-like (XP_042032827.1)	
	YQTGQSEQVYQQAR	2S Albumin-like (XP_042053718)	
Coconut	**EVDEVLNAPR (1)**	Vicilin-like Antimicrobial Peptides 2-2 (KAG1347739.1)	
	GDEVAIFTPR	Cocosin-1 (KAG1354301.1)	
	GFSTELLAAAFGVNMELAR	Cocosin-1 (KAG1354301.1)	[1]
	GLLLPSMSNAPR (3)	Cocosin-1 (XP_019709412.1)	
	**LNALEPTR (2)**	Cocosin-1 (KAG1354301.1)	
	SEAGITDYFDEDNEQFR	11S globulin isoform 2 (AKS26849.1)	
Flaxseed	ANGFDGVANPDIVAR	Chitinase IV (ABA39179.1)	
	ASEFGQQHVTGGQQTS	Oleosin low molecular weight isoform (ABB01618.1)	
	AVAQEQVDK	Late Embryogenesis Abundant Group 1 (AFN53712.1)	
	AVLPQGFGATIR	Chitinase IV (ABA39179.1)	
	**FFLAGNPQR (1)**	*de novo*	
	**LIYVDQGR (2)**	*de novo*	
	MQDAAGYMGQK	Oleosin high molecular weight isoform (ABB01616.1)	[1]
	QDIQQQGQQQEVER	Conlinin (CAC94011.1)	[1]
	QEIQQQGQQQEVQR	Conlinin (CAC94010.1)	
	QIQEQDYLR	Conlinin (CAC94011.1)	[1]
Hemp	EQEGLPNNVFR	Edestin-3 (SNQ45158.1)	
	FYIAGNPHEDFPQSR (3)	11S Globulin (XP_030499769.1)	[2]
	FYLAGNPEDEFEQLR	Edestin-1 (CDP79024.1)	
	GEDLQIIAPSR	Edestin-2 (CDP79027.1)	[2]
	**GTLDLVSPLR (1)**	Edestin-1 (CDP79024.1)	[1,2]
	**ILAESFNVDTELAHK (2)**	Edestin-2 (CDP79027.1)	[2]
	IQSQDDFR	Edestin-3 (SNQ45160.1)	
	YLEEAFNVDSETVK	Edestin-1 (CDP79024.1)	[1,2]
Pumpkin	AFYLAGNPEEEFPER	11S Globulin Subunit Beta (XP_022967669.1)	
	GLLLPQYTNAPR	11S Globulin Subunit Beta (XP_022967669.1)	
	ISNEEALR	11S Globulin Subunit Beta (XP_022967669.1)	
	ISTANYHTLPVLR (3)	11S Globulin Subunit Beta (XP_023000493.1)	[1]
	**LVFVAQGFGIR (1)**	11S Globulin Subunit Beta (XP_023000493.1)	[1]
	VEGELEVIKPPR	11S Globulin Subunit Beta (XP_022995606.1)	
	**VLAEIFNINVETAR (2)**	11S Globulin Subunit Beta-like (XP_022995606.1)	
Rapeseed	AHEAHDTSLTTETR	Oleosin S2-2-like (XP_013677557.1)	[1]
	EFQQAQHLK	Napin-B (XP_013743463.1)	

	FSTLETTLTQSSGPMGYGMPR	Cruciferin Cru4 subunit (CAA40980.1)	[1]
	GSIHNNAMVLPQWNVNANAALYVTK	Cruciferin Cru4 subunit (CAA40980.1)	[1]
	LTFVVHGHALMGK	Cruciferin Cru4 subunit (CAA40980.1)	[1]
	LVIEQGGLYLPTFFSSPK	Cruciferin Cru1-like (XP_013656366.1)	
	**NLRPFLIAGNNPQGQQWLQGR (1)**	Unnamed protein (CAF2310147.1)	
	QQGQQGQQGQQMQQVVSR	Unnamed protein (CAF2150198.1)	
	**QQQGQQGQQLQQVISR (2)**	Napin Large Chain L2B (AAB37417.1)	
	VEYWDHNNPQIR	Cruciferin Cru1 (XP_013696793.1)	
	VGYGAGYDYGYGADYK	Oleosin S2-2-like (XP_013677557.1)	[1]
	VQGPFSVIRPPLR	Cruciferin BnC1 (XP_022557043.1)	
	VQGPFSVLRPPLR	*de novo*	
Rapeseed/ Mustard	**NLRPFLLAGNNPQGQQWLQGR**	Cruciferin Cru4 Subunit (CAA40980.1)	
Sesame	**AFYLAGGVPR (2)**	11S Globulin Seed Storage Protein 2 Precursor (NP_001291336.1)	[3]
	AGNNGFEWVAFK	11S Globulin Seed Storage Protein 2 Precursor (NP_001291336.1)	[1]
	DVANEANQLDLK	Legumin B-like Precursor (NP_001291327.1)	
	FESEAGLTEFWDR	11S Globulin Subunit Beta-like Precursor (NP_001291328.1)	
	GSQSFLLSPGGR	11S Globulin Seed Storage Protein 2 Precursor (NP_001291336.1)	[4]
	ISGAQPSLR (3)	11S Globulin Seed Storage Protein 2 Precursor (NP_001291336.1)	[3]
	IQSEGGTTELWDER	11S Globulin Seed Storage Protein 2 Precursor (NP_001291336.1)	
	**LVLPEYGR (1)**	Legumin B-like Precursor (NP_001291327.1)	
Sunflower	ALPVDVLANAYQLSR	11S Globulin Seed Storage Protein G3-like (XP_021982239.1)	
	FFLAGNPQAQAQSQQQQQR	11S Globulin Seed Storage Protein G3 (XP_021988017.1)	
	FFLAGNPQAQSQQQHR	11S Globulin Seed Storage Protein G3-like (XP_021973262.1)	[1]
	**FPILEHLR (2)**	11S Globulin Seed Storage Protein G3 (XP_021988017.1)	
	FPILEHLQLSADR	11S Globulin Seed Storage Protein G3-like (XP_021993539.1)	
	**FPILEHLQLSAER (1)**	Putative 11-S Seed Storage Protein (KAF5799341.1)	[1]
	LPILQMVQLSAER	11S Globulin Seed Storage Protein 1 (XP_021987363.1)	

1. Kotecka-Majchrzak, K.; Sumara, A.; Fornal, E.; Montowska, M. Proteomic analysis of oilseed cake: a comparative study of species-specific proteins and peptides extracted from ten seed species. *J Sci. Food Agric.* **2021**, *101*, 297–306, doi:10.1002/jsfa.10643.
2. Kotecka-Majchrzak, K.; Kasalka-Czarna, N.; Sumara, A.; Fornal, E.; Montowska, M. Multispecies identification of oilseed- and meat-specific proteins and heat-stable peptide markers in food products. *Molecules* **2021**, *26*, 14, doi:10.3390/molecules26061577.
3. Huschek, G.; Bonick, J.; Lowenstein, Y.; Sievers, S.; Rawel, H. Quantification of allergenic plant traces in baked products by targeted proteomics using isotope marked peptides. *LWT-Food Sci. Technol.* **2016**, *74*, 286–293, doi:10.1016/j.lwt.2016.07.057.
4. Croote, D.; Braslavsky, I.; Quake, S.R. Addressing complex matrix interference improves multiplex food allergen detection by targeted LC-MS/MS. *Anal. Chem.* **2019**, *91*, 9760–9769, doi:10.1021/acs.analchem.9b01388.

Supplementary Table S2. Parameters of scheduled MRM method for alternative peptide markers for the oilseed species (MRM detection window 40s; CE = collision energy; CXP = cell exit potential; DP = declustering potential). The product ions are listed in decreasing intensity.

	Peptide Marker	T_R [Min]	DP [V]	m/z (Charge State)	Product Ions	CE [V]	CXP [V]
Chia 3	NTLRPNALSLPNYHPNPR	4.21	111	519.3 (+4)	483.3 (y4), 620.3 (y5), 497.7 ($y8^{2+}$)	31/19/23	30/20/30
Coconut 3	GLLLPSMSNAPR	5.85	71	628.3 (+2)	397.3 (b4), 430.2 ($y8^{2+}$), 369.3 (a4)	21/27/25	22/18/12
Hemp 3	FYIAGNPHEDFPQSR	4.37	96	593.3 (+3)	734.4 ($y13^{2+}$), 311.1 (b2), 677.8 ($y12^{2+}$)	23/25/25	40/28/40
Peanut 3	GTGNLELVAVR	6.29	96	564.8 (+2)	345.2 (y3), 686.4 (y6), 557.4 (y5)	29/31/33	24/38/28
Pumpkin 3	ISTANYHTLPVLR	4.41	80	495.6 (+3)	686.5 ($y12^{2+}$), 642.9 ($y11^{2+}$), 698.5 (y6)	23/19/31	40/30/44
Sesame 3	ISGAQPSLR	1.04	90	464.8 (+2)	472.3 (y4), 728.4 (y7), 815.4 (y8)	21/23/23	26/42/54
Soy 3	SQSDNFEYVSFK	5.19	31	725.8 (+2)	381.2 (y3), 1235.6 (y10), 643.3 (y5)	35/29/35	26/52/46

Supplementary Table S3. Peptide markers for chia, coconut, flaxseed, hemp, pumpkin, rapeseed, sesame and sunflower and their homologies (NCBI online search tool BLAST, parameters for database search: query cover = 100%, percent identity = 100%; without bacteria); Peptide 1 = quantifier, Peptide 2 = qualifier, Peptide 3 = alternative peptide marker; *Target proteins refer to *Salvia splendens*; **predicted homologies according to the NCBI database, which were not confirmed experimentally. Target proteins of pumpkin refer to *Cucurbita maxima*.

**Marker**	**Peptide sequence**	**Protein**	**Homologies (NCBI)**
Chia 1	GPIVIVEK	11S Globulin-like (XP_042050047)*	*Didymodactylos carnosus*
Chia 2	ELQVIKPPFR	11S Globulin-like (XP_042050047)*	
Chia 3	NTLRPNALSLPNYHPNPR	11S Globulin Seed Storage Protein 2-like (XP_042032827)*	*Perilla frutescens*
Coconut 1	EVDEVLNAPR	Vicilin-like Antimicrobial Peptides 2-2 (KAG1347739)	*Phoenix dactylifera, Elaeis guineensis*
Coconut 2	LNALEPTR	Cocosin-1 (KAG1354301)	*Phoenix dactylifera, Elaeis guineensis*
Coconut 3	GLLLPSMSNAPR	Cocosin-1 (KAG1354301)	*Elaeis guineensis*
Flaxseed 1	FFLAGNPQR		*Actinidia chinensis, Buddleja alternifolia**, Camellia sinensis**, Jatropha curcas**, Nicotiana spp.**, Populus spp., Ricinus communis, Solanum chilense, Solanum commersonii**, Solanum tuberosum**, Solanum lycopersicum**, Solanum pennellii, Telopea speciosissima*
Flaxseed 2	LIYVDQGR		*Populus trichocarpa*
Hemp 1	GTLDLVSPLR	Edestin-1 (CDP79024)	
Hemp 2	ILAESFNVDTELAHK	Edestin-2 (CDP79027)	
Hemp 3	FYIAGNPHEDFPQSR	11S Globulin (XP_030499769)	
Pumpkin 1	VLAEIFNINVETAR	11S Globulin Subunit Beta-like (XP_022995606)	*Cucurbita argyrosperma, Cucurbita pepo, Cucurbita moschata*
Pumpkin 2	LVFVAQGFGIR	11S Globulin Subunit Beta (XP_023000493)	*Cucurbita argyrosperma, Cucurbita pepo, Cucurbita moschata*
Pumpkin 3	ISTANYHTLPVLR	11S Globulin Subunit Beta (XP_023000493)	*Cucurbita argyrosperma, Cucurbita pepo, Cucurbita moschata*
Rapeseed 1	NLRPFLIAGNNPQGQQWLQGR	Unnamed Protein Product (CAF2310147.1)	*Brassica rapa, Arabis nemorensis*
	NLRPFLLAGNNPQGQQWLQGR (Isomer to Rapeseed1)	Cruciferin Cru4 Subunit (CAA40980.1)	*Brassica carinata, Brassica cretica, Brassica oleracea, Brassica rapa, Sinapis alba, Raphanus sativus*
Rapeseed 2	QQQGQQGQQLQQVISR	Napin Large Chain L2B (AAB37417.1)	*Brassica carinata, Brassica cretica, Brassica oleracea, Brassica rapa*
Sesame 1	AFYLAGGVPR	11S Globulin Seed Storage Protein 2 Precursor (XP_011080042)	*Buddleja alternifolia, Handroanthus impetiginosus*
Sesame 2	LVLPEYGR	Legumin B-like Precursor (XP_011091309)	*Clupea harengus, Buddleja alternifolia, Dryobates pubescens*
Sesame 3	ISGAQPSLR	11S Globulin Seed Storage Protein 2 Precursor (XP_011080042)	*Pogona vitticeps*
Sunflower 1	FPILEHLQLSAER	Putative 11-S Seed Storage Protein (KAF5799341)	
Sunflower 2	FPILEHLR	11S Globulin Seed Storage Protein G3 (XP_021988017)	*Brettanomyces bruxellensis, Pleurotus ostreatoroseus, Zostera marina*
Sunflower 3	FPILEHLQLSADR	11S Globulin Seed Storage Protein G3-like (XP_021993539)	

Supplementary Table S4. Groups of possible ingredients for the production of sausages and commercial spice mixtures, which were tested regarding cross-reactivity with the oilseed marker peptides analyzed.

**Group 1: Cereals**	**Group 2: Millet species**
Barley (*Hordeum vulgare*)	Golden millet (*Setaria sphacelata)*
Miaze (*Zea mays*)	Pancile millet (*Panicum miliaceum*)
Oat (*Avena sativa*)	Pearl millet (*Pennisetum glaucum*)
Rice (*Oryza sativa*)	Red colen millet (*Setaria italica*)
Rye (*Secale cereale*)	Silver millet (*Panicum miliaceum*)
Spelt (*Triticum aestivum subsp. spelta*)	Sorghum millet (*Sorghum bicolor*)
Tricitale (*Tscherm.-Seys. ex Müntzing*)	Teff (*Eragrostis tef*)
Wheat (*Triticum aestivum*)	Yellow colen millet (*Setaria italica*)

**Group 3: Nuts**	**Group 4: Turnips**
Almond (*Prunus dulcis*)	Beetroot (*Beta vulgaris*)
Brazil nut (*Bertholletia excelsa*)	Carrot (*Daucus carota*)
Cashew (*Anacardium occidentale*)	Cultivated radish (*Raphanus sativus var. Sativus)*
Hazelnut (*Corylus avellana*)	Horseradish (*Armoracia rusticana*)
Macadamia (*Macadamia spp.*)	Mangold (*Beta vulgaris subsp. vulgaris*)
Pecan nut (*Carya illinoinensis*)	Parsley (*Petroselinum crispum*)
Pine seeds (*Pinus pinea*)	Parsnip (*Pastinaca sativa*)
Pistachio (*Pistacia vera*)	Radish (*Raphanus spp.*)
Walnut (*Juglans regia*)	Turnip (*Brassica napus subsp. rapifera*)

**Group 5: Legumes**	**Group 6: Roots and Tubers**
Brown Lentil (*Lens culinaris Medik.*)	Celery (tuber) (*Apium graveolens*)
Chick pea (*Cicer arietinum*)	Fennel (*Foeniculum vulgare*)
Lupine (blue) (*Lupinus angustifolius*)	Ginger (*Zingiber officinale*)
Lupine (white) (*Lupinus albus*)	Jerusalem artichoke (*Helianthus tuberosus*)
Lupine (yellow) (*Lupinus luteus*)	Manioc (*Manihot esculenta)*
Pea (*Pisum sativum*)	Potato (*Solanum tuberosum*)
Red Lentil (*Lens culinaris.*)	Sweet Potato (*Ipomoea batatas*)

**Group 7: Fruit Vegetables**	**Group 8: Vegetables cabbage**
Avocado (*Persea americana Mill*)	Broccoli (*Brassica oleracea var. italica Plenck*)
Chili (*Capsicum spp.*)	Brussel sprouts (*Brassica oleracea var. gemmifera*)
Cucumber (*Cucumis sativus*)	Cabbage (*Brassica oleracea convar. capitata var. alba*)
Eggplant (*Solanum melongena*)	Cauliflower (*Brassica oleracea var. botrytis*)
Habanero (*Capsicum chinense*)	Kale (*Brassica oleracea var. sabellica*)
Olive (*Olea europaea*)	Kohlrabi (*Brassica oleracea var. gongylodes*)
Paprika (*Capsicum annuum*)	Red cabbage (*Brassica oleracea convar. capitata var. rubra*)
Tomato (*Solanum lycopersicum*)	Savoy cabbage (*Brassica oleracea convar. capitata var. sabauda*)

**Group 9: Other**	**Group 10: Leafy Vegetables**
Asparagus (*Asparagus officinalis*)	Artichoke (*Cynara cardunculus subsp. scolymus)*
Bamboo (*Bambusoideae Luerss.*)	Celery (sheets) (*Apium graveolens*)
Mustard (yellow/white) (*Sinabis alba)*	China cabbage (*Brassica rapa subsp.)*
Mustard (brown) (*Brassica juncea*)	Pak choi *(Brassica rapa subsp. Chinensis*)
Mustard (black) (*Brassica nigra*)	Spinach (*Spinacia oleracea*)
Poppy (*Papaver*)	

**Group 11: Fungi & Algae**	**Group 12: Beans**
Champignon (*Agaricus spp.*)	Black turtle bean (*Phaseolus vulgaris*)
Chanterelle (*Cantharellus cibarius*)	Broad bean (*Vicia faba*)
Oyster mushroom (*Pleurotus ostreatus*)	Garden bean (*Phaseolus vulgaris*)
King trumpet mushroom (*Pleurotus eryngii*)	Jack bean (*Canavalia ensiformis*)
Nori (*Porphyra C. Agardh*)	Kidney bean (*Phaseolus vulgaris*)
Porcino (*Boletus*)	Lima bean (*Phaseolus lunatus*)
Quorn (*Fusarium venenatum*)	Mung bean (*Vigna radiata*)
Sea-Spaghetti (*Himanthalia elongata*)	Navy bean (*Phaseolus vulgaris*)
Shiitake (*Lentinula edodes*)	Pinto bean (*Phaseolus vulgaris*)
	Runner bean (*Phaseolus coccineus*)

**Group 13: Onions & Leeks**	**Group 14: Special fruits & thickeners**
Garlic (*Allium sativum*)	Apple (*Malus Mill.*)
Leek (*Allium ampeloprasum*)	Banana (*Musa × paradisiaca*)
Onion (*Allium cepa*)	Black currant (*Ribes nigrum*)
Shallot (*Allium ascalonicum*)	Carob bean gum (*Ceratonia siliqua*)
Spring onion (*Allium fistulosum*)	Jackfruit (*Artocarpus heterophyllus*)
Wild garlic (*Allium ursinum*)	Orange (*Citrus sinensis*)
	Psyllium husks (*Plantago indica)*

**Group 15: Pseudo cereals**	**Group 16: Spices I**
Amaranth (*Amaranthus*)	Cardamom (*Elettaria cardamomum*)
Buckwheat (*Fagopyrum Mill.*)	Fenugreek (*Trigonella foenum-graecum*)
Quinoa (*Chenopodium quinoa*)	Lovage (*Levisticum officinale*)
	Turmeric (*Curcuma longa*)
	Sage (*Salvia*)

**Group 17: Spices II**	**Group 18: Commercial spice mixtures**
Caraway (*Carum carvi*)	Bavarian veal sausages
Cloves (*Dianthus*)	"Bratwurst"
Coriander (*Coriandrum sativum*)	Blood sausages/"Pressack" red
Mace (*Myristicae arillus*)	Curry (for various sausages)
Majoram (*Origanum majorana*)	Emulsion-type sausages ("Lyoner")
Nutmeg (*Myristica fragrans*)	Frankfurter-type sausages
	"Gelbwurst"
	Ham sausages
	Liver sausage

Supplementary Table S5. Parameters of scheduled MRM method for peptide markers of casein and pork (MRM detection window 40 s; CE = collision energy; CXP = cell exit potential; DP = declustering potential). The product ions are listed in decreasing intensity.

	Peptide Marker	t_R [Min]	DP [V]	m/z (Charge State)	Product Ions	CE [V]	CXP [V]
Casein 1	YLGYLEQLLR	7.65	156	634.4 (+3)	991.6 (y8), 334.2 (b3), 658.4 (y5)	29/31/29	54/16/34
Casein 2	FFVAPFPEVFGK	8.38	116	692.9 (+2)	394.2 (b3), 920.5 (y8), 465.2 (b4)	29/25/23	24/48/22
Mustard	ALPLEVITNAYQISLEEAR	9.53	121	710.7 (+2)	504.2 (y4), 817.a (y7), 617.3 (y5)	25/29/21	30/40/32
Pork 1	SALAHAVQSSR	0.50	90	376.2 (+2)	484.8 ($y9^{2+}$), 392.7 (y7+2), 647.3 (y6)	13/21/21	26/18/32
Pork 2	DTLVSQLSR	4.01	86	509.8 (+2)	590.3 (y5), 330.2 (b3), 689.4 (y6)	21/21/27	32/18/44
